# Deferiprone has anti-inflammatory properties and reduces fibroblast migration *in vitro*

**DOI:** 10.1038/s41598-019-38902-2

**Published:** 2019-02-20

**Authors:** Mahnaz Ramezanpour, Jason L. P. Smith, Mian Li Ooi, Michael Gouzos, Alkis J. Psaltis, P. J. Wormald, Sarah Vreugde

**Affiliations:** 1 Department of Surgery - Otorhinolaryngology Head and Neck Surgery, The Queen Elizabeth Hospital, and the University of Adelaide, Adelaide, South Australia; 2 School of Biology, Faculty of Science and Engineering, Flinders University of South Australia, Adelaide, South Australia

## Abstract

Normal wound healing is a highly regulated and coordinated process. However, tissue injury often results in inflammation with excessive scar tissue formation after 40–70% of operations. Here, we evaluated the effect of the iron chelator deferiprone on inflammation and the migration of primary nasal fibroblasts and primary human nasal epithelial cells (HNECs) *in vitro*. The cytotoxicity of deferiprone was examined by the lactate dehydrogenase assay on primary nasal fibroblasts and air-liquid interface (ALI) cultures of HNECs. Wound closure was observed in scratch assays by using time-lapse confocal scanning laser microscopy. Interleukin-6 (IL-6) and type I and III collagen protein levels were determined by ELISA. Intracellular Reactive Oxygen Species (ROS) activity was measured by utilizing the fluorescent probe H2DCFDA. Deferiprone at 10 mM concentration was non-toxic to primary fibroblasts and HNECs for up to 48 hours application. Deferiprone had significant dose-dependent inhibitory effects on the migration, secreted collagen production and ROS release by primary nasal fibroblasts. Deferiprone blocked Poly (I:C)-induced IL-6 production by HNECs but did not alter their migration in scratch assays. Deferiprone has the potential to limit scar tissue formation and should be considered in future clinical applications.

## Introduction

Scar tissue formation is part of the natural healing process after injury. Tissue repair begins within the first few days of an injury and a variety of cytokines and growth factors are involved in the wound healing processes^1^. In the initial stage of wound healing, thrombin cleaves fibrinogen into fibrin monomers, that act as a scaffold to temporarily seal the bleeding at damaged blood vessels^2^. Neutrophils are then attracted to the wound lesion in response to the degranulation of platelets and the activation of the complement cascade^3^. In the second stage of wound repair (2–7 days), while the number of immune cells and inflammatory cytokines decreases, granulation tissue is formed with keratinocytes and fibroblasts migrating to the injury site. Epithelial cells move in the wound bed and form a thin cell layer to close the wound. At the same time, fibroblasts are attracted from the wound edge or from the bone marrow, proliferate and secrete extracellular matrix (ECM) proteins mainly in the form of collagen to form connective tissue. The last stage of wound repair begins 2–3 weeks after injury and can go for a year or more. In this stage, all of the processes activated after injury decrease and the ECM is gradually replaced by type I collagen, the predominant constituent of the normal human dermis. In the normal wound healing process, the scar fades due to reduction of vascularity and shrinks in size from the contraction of the wound under the influence of myofibroblasts^4–6^. However, in an abnormal fibrous wound healing process, the control of tissue repair and regeneration-regulating mechanisms is lost. Clinically, this response is observed as a hypertrophic scar^7,8^. The distinguishing feature of a hypertrophic scar is the continued proliferation of fibroblasts, with excessive deposition of fibroblast-derived ECM proteins and collagen^9^. Whilst low level inflammation is key to normal wound healing, the formation of adhesions or hypertrophic scars following injury can be exacerbated by pathological processes resulting in excessive inflammation^10^. These processes include infection and hematoma formation and it has been shown that recruitment of neutrophils and macrophages, producing inflammatory cytokines and reactive oxygen species (ROS) followed by fibroblast migration and proliferation into the wound are critical factors in these processes^3,11,12^. Hypertrophic scar formation can negatively impact the outcome of surgery such as abdominal surgery, spinal surgery, vascular surgery and heart surgery. Despite a large number of methods that have been used to reduce surgical scars, the optimal treatment method has not been established. Laser therapy^13^, intralesional interferon^14^, silicone gel sheeting^15^, intralesional corticosteroids^16^, pressure therapy^17^, bleomycin^18^ and onion extract gel^19^ are recommended treatments for hypertrophic scar formation which among them, silicone gel, corticosteroids and pressure garments are most common. Silicone gel sheets can be effective in limiting the hypertrophic growth of scars, nevertheless, patients may complain of skin rash, pruritus and excessive sweating^20,21^. Some studies claimed that pressure therapy may prevent scar formation by suppressing collagen production via limiting the supply of nutrients, oxygen and blood to the scar tissue^22^. Other studies found that there was no significant difference between treatments involving the use of high-pressure garments, lower-pressure garments, or no pressure at all^23^. Intralesional corticosteroid injections are second-line therapies for the treatment of hypertrophic scars. Corticosteroids inhibit the inflammatory process and expression of genes related to collagen and glycosaminoglycan synthesis, decreasing fibroblast proliferation. Intralesional steroid injections are highly responsive (50% to 100%), indicating a profound effect of reducing inflammation on limiting hypertrophic scar formation^19,24,25^. However, 63% of the patients experience side effects, especially in the form of hypopigmentation, skin and subcutaneous fat atrophy and some experience telangiectasia^26^.

Deferiprone is an iron chelator with anti-microbial properties^27^ that also has properties of free radical scavenging and is known to improve wound healing (skin wounds)^28^. Scavenging ROS after abdominal surgery has been shown to significantly inhibit postoperative adhesion formation^29^.

This study evaluated the wound-healing activities of different concentrations of deferiprone on primary human fibroblasts and primary human nasal epithelial cells in air liquid interface culture (HNEC-ALI). HNEC-ALI closely mimic the air-facing sinonasal epithelium and produce reliable results when investigating the human sinonasal innate immune system^30^. The goal was to determine the effect of deferiprone on fibroblast and epithelial cell migration, collagen production, ROS activity and potential for anti-inflammatory effects to evaluate its potential to limit hypertrophic scar tissue formation for future clinical applications.

## Results

### *In vitro* cytotoxicity of Deferiprone

**Figure 1:**
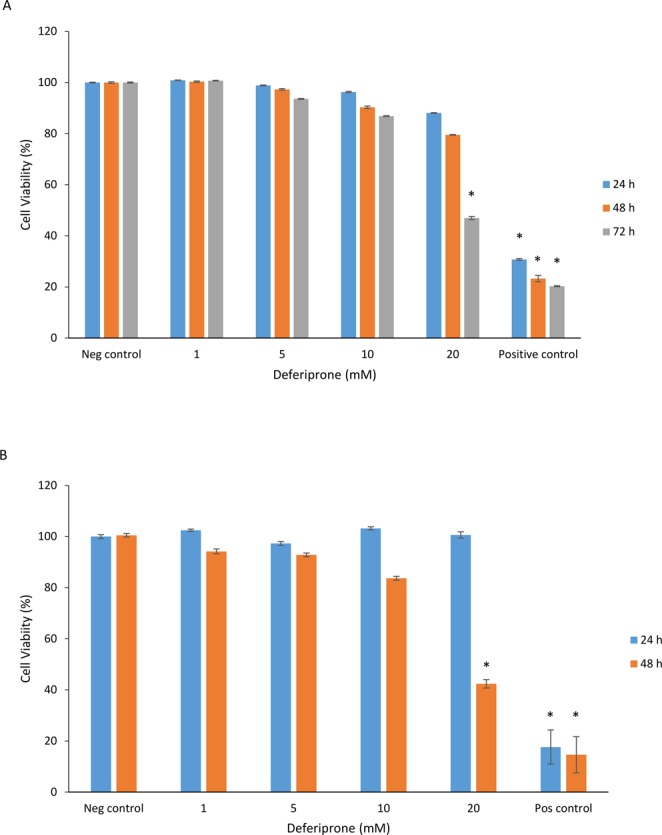
Cell viability of HNECs and human nasal fibroblast monolayers derived from CRS patients. Viability relative to no treatment control cells as determined by the LDH assay, 72 h and 48 hours after application of deferiprone (1 mM, 5 mM, 10 mM, 20 mM), negative control (medium), and positive control (0.5% Triton X-100) in HNECs (**A**) and primary human nasal fibroblasts (**B**) derived from CRS patients. Cell viability was calculated relative to the untreated cells as negative control. The values are shown as means ± SEM, n = 3. ANOVA, followed by Tukey HSD post hoc test. (*p < 0.05).

The cytotoxic effect of different concentrations of deferiprone (1 mM, 5 mM, 10 mM, 20 mM) was determined by the LDH assay, evaluating the survival of HNECs for up to 72 hours (Fig. 1A) and fibroblasts for up to 48 hours (Fig. 1B) relative to the negative control. Concentrations up to 10 mM deferiprone did not show any significant increase in LDH release at different time points in HNECs or fibroblasts (p > 0.05) (Fig. 1A). However, 20 mM showed significant reduction of cell viability after 72 hr and 48 hr in HNECs and fibroblasts respectively (Fig. 1B). The positive control (0.5% Triton X-100) and negative control (medium) demonstrated expected toxicity values.

### Effect of deferiprone on human nasal epithelial cell and primary fibroblast cell migration *in vitro*

**Figure 2:**
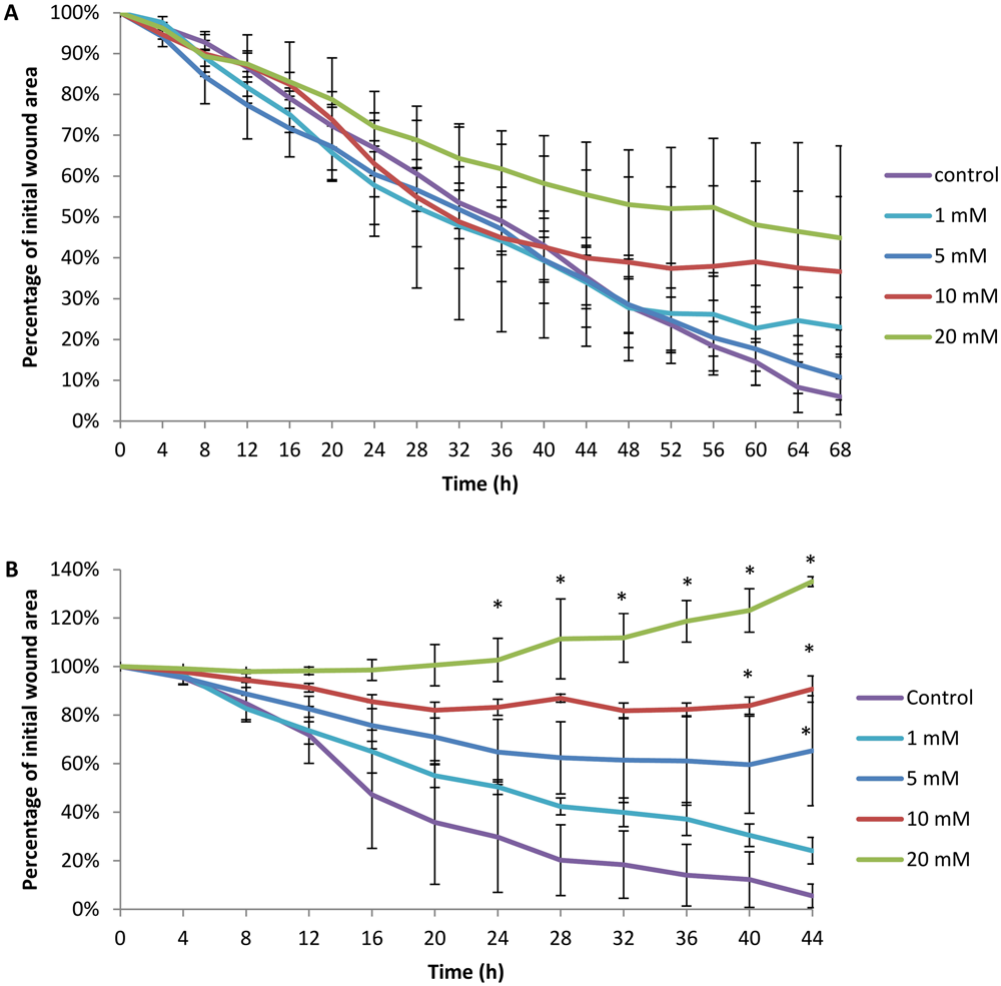
Scratch assays of primary human nasal epithelial cells and primary fibroblasts in the presence of different deferiprone concentrations over time. The mean percentage of wound area in scratch assays of primary human nasal epithelial cells (**A**) and sinonasal fibroblasts (**B**) in the presence of different concentrations of deferiprone (1 mM, 5 mM, 10 mM, 20 mM) or negative (medium) control over time. The values are shown as mean ± SEM, n = 3. ANOVA, followed by Tukey HSD post hoc test. *p < 0.05.

To examine the influence of deferiprone on sinonasal wound resealing *in vitro*, time course studies were performed during active wound closure. HNEC-ALI cultures and primary fibroblasts were treated with different concentrations of deferiprone or negative control in scratch assays. In HNEC-ALI cultures, untreated (control) wounds healed with full re-epithelialization by 68 hours. Incubation with four different concentrations of deferiprone for up to 68 hours did not show any significant delay in HNEC wound healing (Fig. 2A). Untreated primary fibroblasts closed the wound after 44 hours. Incubation with 5, 10 and 20 mM deferiprone significantly delayed fibroblast migration after 44 hours (Fig. 2B).

### Effect of deferiprone on the inflammatory response in human nasal epithelial cells and human primary fibroblasts

**Figure 3:**
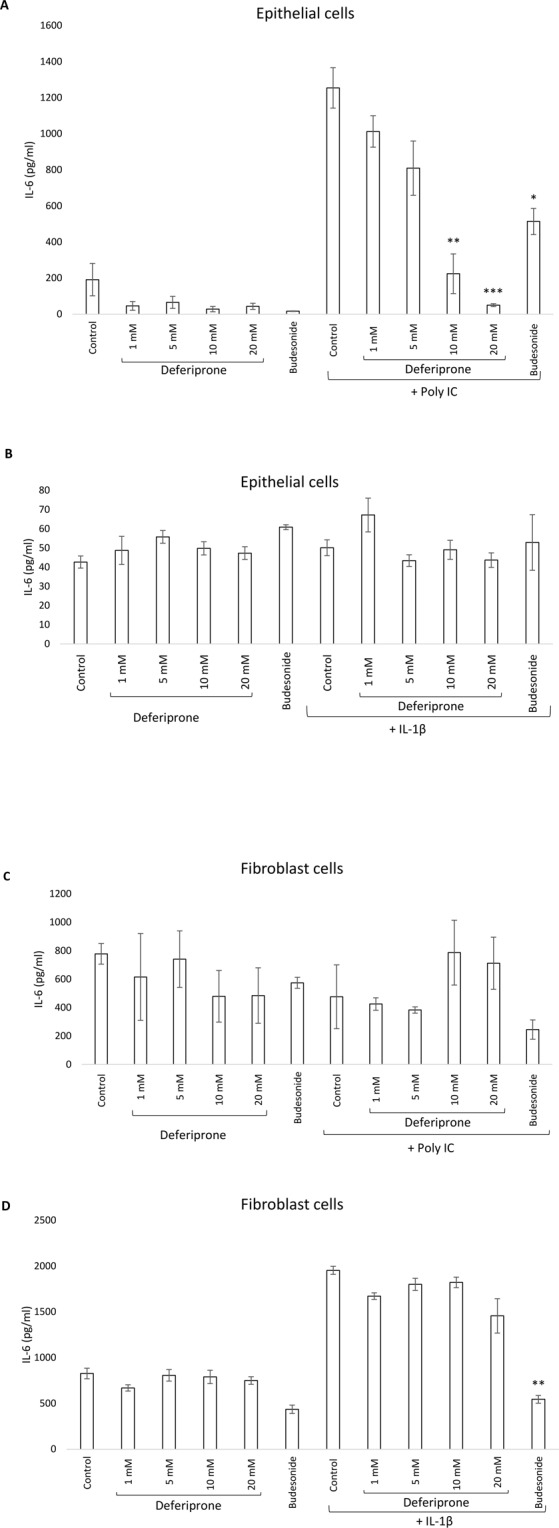
IL-6 production was measured using ELISA on human nasal epithelial cells (**A**,**B**) or nasal fibroblast cells (**C**,**D**) in the presence or absence of the pro-inflammatory agent Poly (I:C) or IL-1β. Budesonide (1 mg/2 ml) was used as anti-inflammatory standard of care control and medium was used as negative control. ANOVA, followed by Tukey HSD post hoc test. (*p < 0.05, **p < 0.001, ***p < 0.0001); values are shown as means ± SEM, n = 4.

To determine the potential of deferiprone to dampen a pro-inflammatory response, deferiprone at different concentrations was applied to HNECs or fibroblasts in the presence or absence of the pro-inflammatory agent Poly (I:C) Low Molecular Weight (LMW) or IL-1β. Budesonide was used as an anti-inflammatory standard of care control and significantly reduced IL-6 production in both HNECs (p = 0.03) and fibroblasts (p = 0.001) in the presence of pro-inflammatory agents. In HNECs, application of 10 mM and 20 mM of deferiprone for 24 hours significantly reduced IL-6 protein concentrations (80% reduction, p = 0.001 and 96% reduction, p = 0.0001 respectively) in the presence of Poly (I:C) (Fig. 3A) compared with negative control. In contrast, deferiprone did not alter the secretion of IL-6 in nasal fibroblasts in the presence or absence of IL-1β after 24 hours (Fig. 3D).

### Effect of deferiprone on the release of collagen by human primary fibroblasts

**Figure 4:**
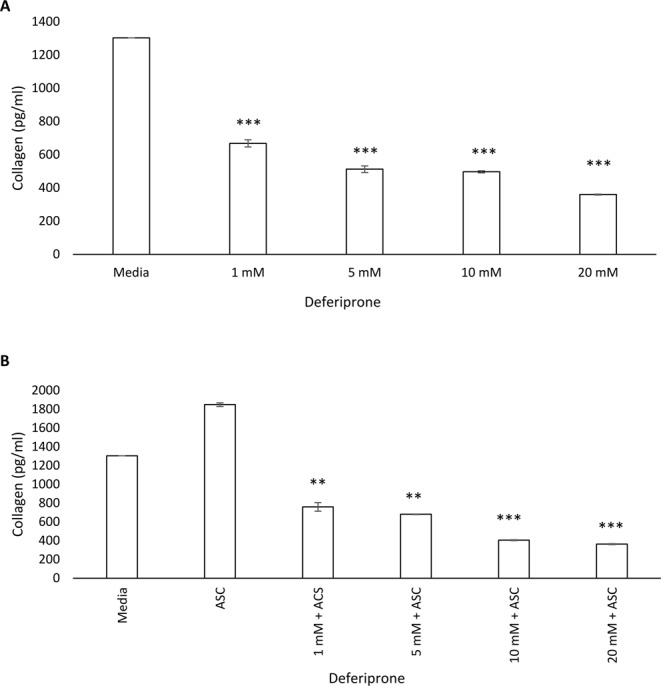
Collagen release was measured by ELISA in primary nasal fibroblasts treated with deferiprone in the absence (**A**) or presence (**B**) of L-Ascorbic acid-2 phosphate (ASC). Primary human nasal fibroblasts were treated with 1 mM, 5 mM, 10 mM and 20 mM deferiprone for 24 hours. Media only and L-Ascorbic acid-2 phosphate (ASC) acted as a negative and positive control respectively. Bars stand as means ± standard deviation (n = 4). (**p < 0.001, ***p < 0.0001). ANOVA, followed by Tukey HSD post hoc test.

Application of different concentrations of deferiprone for 24 hours significantly reduced collagen type I protein concentrations in supernatants of fibroblast monolayers derived from CRS patients (*p* < 0.0001) (Fig. 4A). In addition, deferiprone at different concentrations was applied to fibroblasts in the presence of L-Ascorbic acid-2 phosphate (ASC), known to induce collagen production by fibroblasts^31^. Deferiprone significantly inhibited collagen type I secretion in the presence ASC (Fig. 4B). No type III collagen was found in the supernatants of fibroblasts in the presence/absence of different concentrations of deferiprone (Data not shown).

### Effect of deferiprone on the release of reactive oxygen species in human nasal epithelial cells and human primary fibroblasts

**Figure 5:**
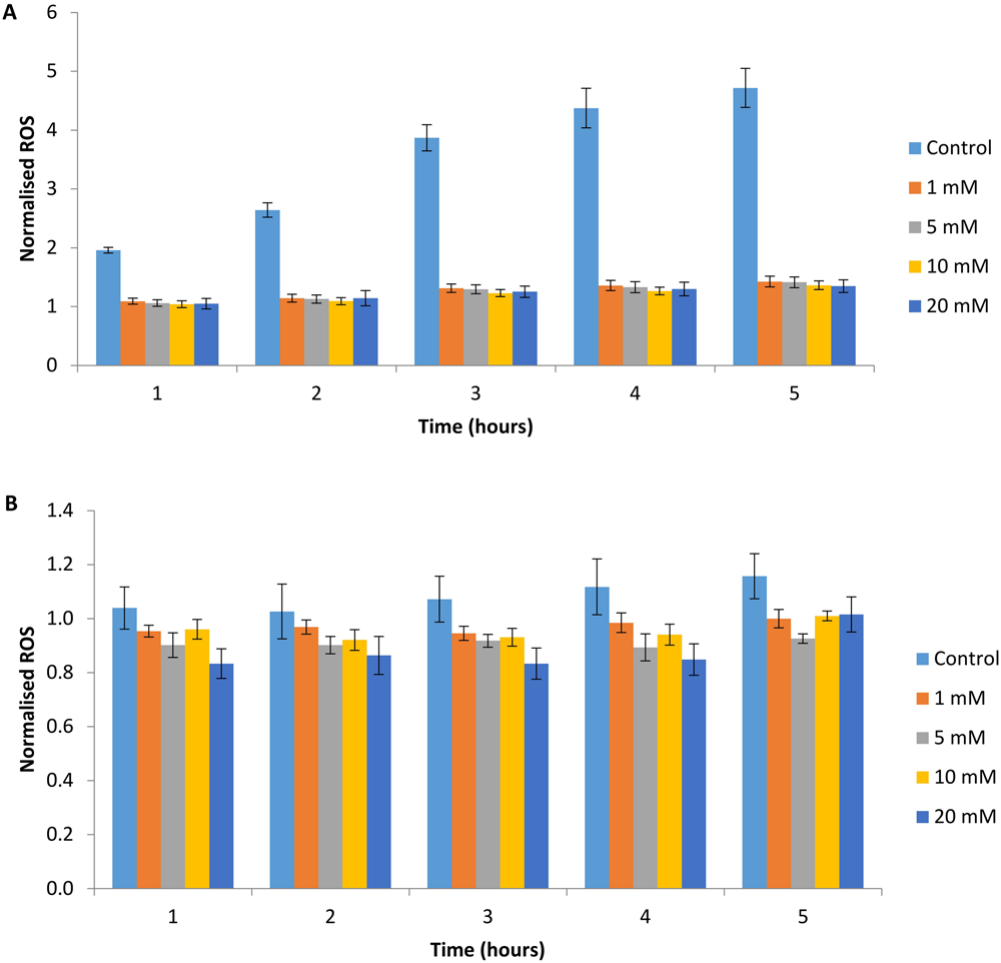
ROS quantification by measuring the dichlorofuorescein diacetate (H2DCFDA) probe activation through ROS generation in primary human nasal epithelial cells (**A**) and nasal fibroblast monolayers. (**B**) Data is expressed as ROS accumulation relative to time 0. Statistical analysis was performed by Anova tukey post-hoc for HNECs and Pearson product-moment correlation for fibroblasts, n = 4.

We then measured reactive oxygen species (ROS) production by utilizing the fluorescent probe H2DCFDA. This compound accumulates inside the cells and is oxidized by ROS to the corresponding fluorescent chromophore. The fluorescence intensity was measured using a microplate reader at filter range Ex/Em: 492/525 nm at 1 hr intervals over 5 hr. HNECs showed significant reduction of ROS release with all doses of deferiprone (1 mM, 5 mM, 10 mM, 20 mM) compared with untreated cells (control) (p < 0.001) (Fig. 5A). In addition, There was a significant negative correlation between deferiprone dose and ROS release in scratch assays in fibroblast cells at all-time points measured (Fig. 5B). The Pearson product-moment correlation for each time point (except 5 hr) was significant and showed the following correlations: 1 hr, correlation = −0.32; 2 hr, correlation = −0.33; 3 hr, correlation = −0.35, 4 hr, correlation = −0.44 (Fig. 5B).

## Discussion

In this study on the effect of deferiprone on wound healing activities, we made five fundamental observations. First, deferiprone up to 10 mM was non-toxic when applied onto primary fibroblasts and HNECs *in vitro*. Second, deferiprone significantly delayed fibroblast migration in a dose-dependent way when compared with untreated samples. In contrast, HNECs did not show any significant change in their migration rate after deferiprone treatment. Third, deferiprone blocked IL-6 production in pro-inflammatory conditions in HNECs but not in fibroblasts. Fourth, deferiprone reduced collagen protein concentrations in the supernatants of fibroblast monolayers in the presence or absence of L-Ascorbic acid-2 phosphate (ASC). Finally, deferiprone inhibited ROS release in human nasal epithelial cells and human sinonasal fibroblasts compared with untreated samples. The absence of toxicity of deferiprone (1 mM, 5 mM and 10 mM) is in line with our previous reports where treatment with deferiprone (alone or in combination with other compounds) showed no significant toxic effects *in vitro* when applied to L929 and NuLi-1 cell lines^27^ and *in vivo* when applied to the sinus mucosa of sheep^32^. It has been demonstrated that the critical time interval to block adhesions is primarily in the first few days after the initial injury and that the extent of adhesion formation is largely dependent on the level of inflammation, ROS production, collagen production and fibroblast migration during that time^10,33^. Here, the 44 h exposure to 5 mM, 10 mM and 20 mM deferiprone caused a significant delay in fibroblast migration, whereas HNECs re-epithelialized at the same rate as the untreated control. In fact, application of 20 mM deferiprone to fibroblasts increased the percentage of initial wound area above 100%. This might be due to the reduced cell viability observed for this concentration after prolonged continuous exposure. Our data implies that deferiprone might be helpful in treating post-surgical adhesions as it limits fibroblast cell-adhesions without negatively affecting the re-epithelialization. Fibroblasts and smooth muscle cells are responsible for collagen synthesis and any factor that decreases collagen synthesis results in a longer-lasting wound healing. Our data indicate that stimulation of the fibroblasts with deferiprone decreased type I collagen production in a dose-dependent manner. For further scrutiny, we treated the fibroblasts in the presence of ASC which is known to stimulate collagen accumulation and cell proliferation^31^. Interestingly, we found deferiprone significantly inhibited collagen secretion even in the presence of ASC. Consistent with our finding, deferiprone has been shown to inhibit the proliferation of skin fibroblasts and frataxin- depleted neuroblastoma-derived cells *in vitro*^34^. The study showed that deferiprone impaired aconitase activity through reduced synthesis of the iron-sulfur cluster machinery which makes it a potent chelator of mitochondrial matrix iron^34^.

Balanced inflammation is critical to the normal healing process after tissue injury. However, excessive inflammation can result in hypertrophic scar formation and chronic inflammation is a critical factor in delaying the wound healing of chronic wounds^10,35^. Critical inflammatory cells involved in this process are neutrophils and macrophages^10^ and their recruitment is associated with the induction of ROS and inflammatory cytokine production^36^. In an *in vitro* wound model of hypertrophic scar fibroblasts, a microarray analysis indicated the interleukin 6 (IL-6) signaling pathway to be the main pathway involved in the early response to injury in those cells^37^. Moreover, IL-6, a key chemoattractant for monocytes and a macrophage activator, along with other proinflammatory factors, such as interleukin (IL)-1α, IL-1β and tumor necrosis factor-α are upregulated in hypertrophic scar tissues^30,35^. Moreover, decreased levels of IL-6 characterize foetal wounds, known to heal without scarring, and the addition of IL-6 to foetal wounds leads to scarring^38^.

Together these findings indicate that limiting inflammation and specifically proinflammatory cytokines such as IL-6 and ROS production, as well as inhibiting the migration of fibroblasts and limiting their collagen production might be key to limiting hypertrophic scar formation. Our findings indicate that deferiprone has the potential to do just that as, in addition to reducing the migration of fibroblasts into the wound and decreasing their collagen production, deferiprone manifestly reduced IL-6 protein production by HNECs in pro-inflammatory conditions and decreased ROS in a dose-dependent manner in fibroblasts.

In conclusion, the results of this study indicate that deferiprone was not toxic to primary fibroblasts or HNECs. Deferiprone, in a dose and time-dependent way, delayed primary nasal fibroblast migration in scratch assays, decreased their collagen and ROS production and reduced immune cytokine IL-6 production by HNECs. Together, our observations indicate that deferiprone may have the potential to limit scar tissue formation in future clinical applications.

## Materials and Methods

### Study population

This study was performed in accordance with guidelines approved by the Human Ethics Committee of the Queen Elizabeth Hospital and the University of Adelaide. All patients gave written informed consent (reference HREC/15/TQEH/132) and all samples obtained were anonymised and coded before use. All methods were carried out in accordance with the relevant guidelines and regulations. Patients recruited to the study included those who were undergoing endoscopic sinus surgery for chronic rhinosinusitis (CRS). Exclusion criteria included active smoking, age less than 18 years, pregnancy, and systemic diseases (immunosuppressive disease).

### Harvesting and culturing primary Human Nasal Fibroblasts *in Vitro*

Sinonasal tissue was biopsied from paranasal sinus mucosa and transferred to a 6-well culture plate with 2 ml Dulbecco’s Modified Eagle’s medium (DMEM, Invitrogen, UK) supplemented with L-glutamine, 10% Fetal bovine serum (FBS, Sigma, St Louis, USA) and penicillin streptomycin (Gibco, Life Technologies, NY, USA). Every 2–3 days, the tissue was washed gently with 1 ml phosphate-buffered saline (PBS) and medium was replaced with 1.5 ml fresh medium until fibroblasts became confluent after approximately 2 weeks.

### Purification of fibroblasts

Once confluent, fibroblasts (2 females, 2 males, aged 30–50 years, non-allergic) were washed with 2 ml PBS, trypsinized and collected followed by centrifugation at 400 × *g* for 8 minutes. The supernatant was removed and pellet resuspended in 1 ml PBS along with 50 µl Dynabeads Epithelial Enrich (Invitrogen, NY, USA). The tube was wrapped in parafilm and placed on a rotor mixer for 20 minutes at room temperature (RT). Supernatant containing fibroblasts were transferred to a T25 tissue culture flask (Nunc, Roskilde, Denmark) and the tube containing the remaining beads discarded.

### Harvesting and Culturing Human Nasal Epithelial Cells *in Vitro*

Primary human nasal epithelial cells (HNECs) (3 males, 1 female, non-smoker, aged 45–65 years, non-allergic) were harvested from nasal polyps by gentle brushing in a method described by Ramezanpour *et al*.^39^. Extracted cells were suspended in Bronchial Epithelial Growth Media (BEGM, CC-3170, Lonza, Walkersville, MD, USA), supplemented with 2% Ultroser G (Pall Corporation, Port Washington, NY, USA). The cell suspension was depleted of macrophages using anti-CD68 (Dako, Glostrup, Denmark) coated culture dishes, and HNECs were maintained with B-ALI™ growth medium (Lonza, Walkersville, USA) in collagen coated flasks (Thermo Scientific, Walthman, MA, USA) in a cell incubator at 37 °C with 5% CO_2_.

### Air Liquid Interface Culture

HNECs were grown until 80% confluent then harvested for seeding onto collagen coated 6.5 mm permeable Transwell plates (BD Biosciences, San Jose, California, USA) at a density of 5 × 10^4^ cells per well. Cells were maintained with B-ALI™ growth medium for 2–3 days in a cell incubator at 37 °C with 5% CO_2_. On day 3 after seeding, the apical media was removed and the basal media replaced with B-ALI™ differentiation media, exposing the apical cell surface to the atmosphere. Human nasal epithelial cultures at air liquid interface (HNEC-ALI) were maintained for a minimum of 21 days for development of tight junctions.

### Cytotoxicity Studies

Primary human fibroblasts or HNECs were grown in DMEM and BEGM (Lonza, Walkersville, USA) medium respectively. Cells were maintained in a fully humidified incubator with 5% CO_2_ at 37 °C prior to cytotoxicity studies. Cells were exposed to different concentrations of Deferiprone (3-Hydroxy-1,2-dimethyl-4 (1 H)-pyridone, Sigma, USA) at different time points, followed by determination of lactate dehydrogenase (LDH) with a cytotoxicity detection kit (Promega, Madison, U.S.). Briefly, 50 μL of the supernatant from each well was mixed with 50 μL of LDH reagent and was incubated for 30 minutes in the dark at RT. The optical density (OD) was measured at 490 nm on a FLUOstar OPTIMA plate reader (BMG Labtech, Ortenberg, Germany). Cell culture studies were performed as three independent experiments.

### Wound Healing (Migration) Assay

In the fibroblast wound closure assay, fibroblasts were seeded in 24 well plates, stained with CellTrace™ Violet (Invitrogen/Life Technologies, USA) and allowed to reach 80% confluence in 24 hours. A straight vertical scratch was made down through the fibroblasts and HNEC-ALI cell monolayers by using a 200 μl pipette tip. The media and cell debris was aspirated carefully and culture media with different concentrations of deferiprone (1 mM, 5 mM, 10 mM, 20 mM) or media only (negative control) added to each well for 72 hours. At time zero, cells were treated with 1 µg/ml mitomycin (Accord Healthcare Inc, NDC 16729-108-11, USA) to inhibit cell proliferation. The wound closure (cell migration) was recorded using time-lapse LSM700 confocal scanning laser microscopy (Zeiss Microscopy, Germany), with an image recorded every 4 hours in a temperature and CO_2_ controlled chamber.

### Enzyme-Linked Immunosorbent Assay (ELISA)

Supernatants were collected from HNECs and fibroblasts after 24 hours of exposure with different concentrations of deferiprone in the presence/absence of the pro-inflammatory agent Poly (I:C) (10 µg/ml)^40^ or IL-1β (10 ng/ml Sigma, Saint Louis, USA)^41^ respectively. Interleukin-6 (IL-6) protein levels were estimated with an ELISA kit using rat anti-human IL-6 antibodies (BD Biosciences, New Jersey, USA), according to the manufacturer’s instructions. All measurements were performed in duplicate using a FLUOstar OPTIMA plate reader (BMG Labtech, Ortenberg, Germany). The tissue sample concentration was calculated from a standard curve and corrected for protein concentration.

### Collagen ELISA Assay

Primary human nasal fibroblasts were seeded in 24-well tissue-culture plates at a density of 5 × 10^5^ cells/well grown in DMEM until confluent. Duplicate wells were stimulated with deferiprone at 1 mM, 5 mM, 10 mM and 20 mM in DMEM in the presence/absence of L-Ascorbic acid-2 phosphate (100 mM) (113170-55-1, Sigma-Aldrich) for 48 hours. Following treatment, the supernatant was collected and the protein level of type I C-peptide (Takara Bio Inc, Otsu, Japan)) or type III collagen (LS-F5217, LSBio, WA, USA) was measured with sandwich ELISA Kit. Experimental procedures followed the manufacturer’s instructions. Briefly, 20 μl of culture medium and 100 μl of the antibody-POD conjugate solution were sequentially added into microtiter plates and reacted for 3 hours at 37 °C. After 4× washing with washing buffer solution (1 × PBST), 100 μl of the substrate solution was added and incubated for 15 minutes at RT. Finally, the stop solution (100 μl) was added and corresponding absorbance was recorded at 450 nm using a FLUOstar OPTIMA plate reader (BMG Labtech, Ortenberg, Germany).

### Evaluation of oxidative stress

Primary nasal fibroblast cells were cultured in DMEM with 10% FBS and seeded into black wall 96-well plates (Life Technologies, Australia) and incubated for 24 hr in a humidified incubator with 5% CO_2_ at 37 °C. Cells were washed with PBS and 10 μM of 2′, 7′-dichlorodihydrofluorescein diacetate (H2DCFDA; Invitrogen Life Technologies, Carlsbad, CA, USA) was added for 1 hr, at 37 °C in the dark. Cells were then washed twice with PBS and exposed to scratching injury by dragging a 100 μL pipette tip linearly on the confluent monolayers in the presence of different deferiprone concentrations (1 mM, 5 mM, 10 mM and 20 mM). The fluorescence intensity was then measured using a microplate reader at filter range Ex/Em: 492/525 nm at 1 hr intervals over 5 hr.

### Statistical analysis

A repeated-measures ANOVA was used for statistical analysis of the wound closure. Data is presented as the mean ± SEM. Statistical analyses of LDH assay and ELISA assays were carried out using ANOVA, followed by Tukey HSD post hoc test. These tests were performed using SPSS software (version 22). The Pearson product-moment correlation coefficient was determined by using R software to find correlations of deferiprone dosages with OD values in the oxidative stress assay in fibroblast. Statistical significance was defined as a P value of less than 0.05.
